# Long-term maternal effect on offspring immune response in song sparrows *Melospiza melodia*

**DOI:** 10.1098/rsbl.2006.0544

**Published:** 2006-09-26

**Authors:** Jane M Reid, Peter Arcese, Lukas F Keller, Dennis Hasselquist

**Affiliations:** 1 School of Biological Sciences, Zoology Building, University of AberdeenTillydrone Avenue, Aberdeen AB24 2TZ, UK; 2 Centre for Applied Conservation Research, Forest Sciences, University of British Columbia2424 Main Mall, Vancouver BC, Canada V6T 1Z4; 3 Zoologisches Museum, Universität ZürichWinterthurerstrasse 190, 8057 Zürich, Switzerland; 4 Department of Animal Ecology, Lund UniversityEcology Building, 223 62 Lund, Sweden

**Keywords:** indirect environmental effect, immunocompetence, parasite-mediated selection, tetanus vaccine

## Abstract

Knowledge of the causes of variation in host immunity to parasitic infection and the time-scales over which variation persists, is integral to predicting the evolutionary and epidemiological consequences of host–parasite interactions. It is clear that offspring immunity can be influenced by parental immune experience, for example, reflecting transfer of antibodies from mothers to young offspring. However, it is less clear whether such parental effects persist or have functional consequences over longer time-scales, linking a parent's previous immune experience to future immune responsiveness in fully grown offspring. We used free-living song sparrows (*Melospiza melodia*) to quantify long-term effects of parental immune experience on offspring immune response. We experimentally vaccinated parents with a novel antigen and tested whether parental vaccination influenced the humoral antibody response mounted by fully grown offspring hatched the following year. Parental vaccination did not influence offspring baseline antibody titres. However, offspring of vaccinated mothers mounted substantially stronger antibody responses than offspring of unvaccinated mothers. Antibody responses did not differ between offspring of vaccinated and unvaccinated fathers. These data demonstrate substantial long-term effects of maternal immune experience on the humoral immune response of fully grown offspring in free-living birds.

## 1. Introduction

Variation in host immunity to parasitic infection is thought to underpin major evolutionary processes, including host–parasite coevolution, inter-sexual selection and the evolution of sex, and to influence host and parasite population dynamics (Hamilton & Zuk 1982; May & Anderson 1983; Hamilton *et al*. 1990; Sorci *et al*. 1997). Detailed knowledge of the magnitude and causes of variation in host immunity is therefore integral to predicting the evolutionary and epidemiological consequences of parasite-mediated selection (Little 2002; Grindstaff *et al*. 2003).

Initial immunity depends on additive and non-additive components of host genotype and on an individual's nutritional and hormonal state (Sorci *et al*. 1997; Roitt *et al*. 1998; Reid *et al*. 2003; Owen-Ashley *et al*. 2004). In addition, it is increasingly clear that offspring immunity can also be influenced by parental immune experience (Lemke & Lange 1999; Grindstaff *et al*. 2003; Mitchell & Read 2005). Since such inter-generational phenotypic effects can influence population and evolutionary dynamics (Wolf *et al*. 1998; Beckerman *et al*. 2002), it is pertinent to quantify the magnitude of parental effects on offspring immunity and the time-scale over which these effects act (Grindstaff *et al*. 2003).

In birds, mothers can deposit antibodies and other precursors of immunity in their eggs (Gasparini *et al*. 2001; Grindstaff *et al*. 2003). These maternal effects have been quantified over short time-scales, for example, by measuring maternal antibodies in eggs or young chicks following present or recent maternal exposure to specific immune challenges (Gasparini *et al*. 2001; Müller *et al*. 2004; Grindstaff *et al*. 2006). However, it is less clear whether parental effects persist over longer time-scales, in terms of the time between parental exposure and reproduction, or the lasting influence of parental exposure on immunity in fully grown offspring. Furthermore, few studies have tested whether parental immune experience influences offspring immune responsiveness rather than solely baseline antibody titres; quantifying some measure of functional immunity may better indicate the fitness consequences of parentally derived immunity (Heeb *et al*. 1998; Gasparini *et al*. 2002; Grindstaff *et al*. 2003). Further data describing the long-term impact of parental immune experience on offspring immune response are therefore required. Such data should ideally be collected from free-living individuals to ensure that parental effects are quantified in comparison with realistic direct environmental and genetic influences on offspring immunity (Sorci *et al*. 1997).

We used free-living song sparrows (*Melospiza melodia*) to test whether parental exposure to a novel antigen influenced the future humoral immune response of fully grown offspring. During September 2004, we vaccinated a sample of song sparrows with tetanus toxoid. Twelve months later, we tested whether baseline tetanus antibody titre or primary antibody response differed between fully grown offspring of vaccinated and unvaccinated parents. Our use of a novel antigen to assess humoral immunity allows us to distinguish long-term effects of parental vaccination from effects of subsequent natural parasite exposure on offspring immune response (Gasparini *et al*. 2002; Staszewski & Boulinier 2004).

## 2. Material and methods

The resident population of song sparrows on Mandarte Island, Canada, has been studied intensively since 1975. Each year, all fledgling song sparrows are individually colour-ringed before leaving their natal territory. Each individual's life history is then documented, allowing parents and offspring to be identified across years (Smith *et al*. 2006). Song sparrows typically breed two to three times per year, during March–July. Offspring become independent from their parents within 30 days of hatching (Smith *et al*. 2006).

During September 2004, we mist-netted song sparrows on Mandarte as part of a wider study of individual variation in immune response. Captured individuals were vaccinated with 70 μl of tetanus toxoid in the pectoral muscle and released (*n*=49) or released without vaccination (*n*=28). Approximately 40 further colour-ringed individuals were observed but not captured. Then, during September 2005, we measured baseline tetanus antibody titres and the primary antibody response to tetanus vaccination in adult song sparrows and their fully grown offspring that had hatched during 2005 (see electronic supplementary material). Briefly, sparrows were mist-netted, blood sampled, vaccinated with 70 μl of tetanus toxoid and released. Individuals were recaptured and blood sampled *ca* 10 days later. Tetanus antibody titres in baseline and post-vaccination plasma samples were quantified by enzyme-linked immunosorbent assay. Tetanus response was estimated as the difference between post-vaccination and baseline antibody titres. Fieldwork was approved by the University of British Columbia Animal Care Committee.

We used general linear mixed models to test whether an offspring's baseline antibody titre or tetanus response, measured in September 2005, depended on whether its parents had been vaccinated in September 2004. We controlled for offspring sex, offspring and parental inbreeding coefficients (*f*), and the period between baseline and post-vaccination blood samples, since these variables influence tetanus response in song sparrows (Reid *et al*. submitted). Since samples included sets of siblings, we initially modelled ‘family’ as a random factor. Antibody data were log-transformed to reduce deviations from normality. Analyses were run in SPSS (v. 14.0) and R (v. 2.2.1). All tests were two-tailed. Variables were retained if *p*≤0.1. All interaction terms were eliminated. Means are presented ±1 s.e.

## 3. Results

In September 2005, baseline and post-vaccination tetanus antibody titres were measured in 46 fully grown offspring of 21 mothers and 23 fathers (comprising 23 pairings). Eleven mothers (of 21 offspring) and 10 fathers (of 19 offspring) had been vaccinated in 2004. Ten mothers (of 25 offspring) and 13 fathers (of 27 offspring) had not been vaccinated. Vaccinated and unvaccinated parents did not differ with respect to *f*, age or reproduction in 2005 (table 1).

**Figure 1:**
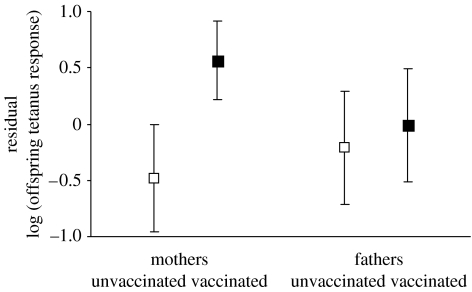
Residual (log) tetanus responses (controlling for offspring *f*, paternal *f* and inter-sample period) of offspring of mothers and fathers that had and had not been vaccinated with tetanus toxoid 12 months previously (filled and open symbols, respectively). Means and 95% confidence limits are shown.

Across all 46 offspring, baseline antibody titres averaged 7.2±0.7 units and did not differ between offspring whose parents had and had not been vaccinated the previous year (table 2). Tetanus response averaged 222.6±35.7 units. Offspring of vaccinated mothers mounted greater tetanus responses than those of unvaccinated mothers (table 2, figure 1, predicted responses were approximately 350 and 90 units, respectively); maternal vaccination explained 23% of variation in offspring tetanus response. Tetanus response did not differ between offspring of vaccinated and unvaccinated fathers (table 2, figure 1).

## 4. Discussion

Previous studies have demonstrated increased antibody titres in eggs or young chicks of parents exposed to specific immune challenges (Gasparini *et al*. 2001; Grindstaff *et al*. 2003, but see Lozano & Ydenberg 2002). In song sparrows, baseline tetanus antibody titres did not differ between offspring whose parents had and had not been vaccinated the previous year. This absence of a long-term effect of parental vaccination on baseline antibody titres in fully grown offspring is not surprising, since parental antibodies are catabolized within weeks and vertebrates do not generally synthesize specific antibodies in the absence of relevant antigens (Roitt *et al*. 1998; Grindstaff *et al*. 2003). However, when fully grown offspring were themselves vaccinated with tetanus toxoid, offspring of mothers that had been vaccinated 12 months earlier (and seven to nine months prior to reproduction) mounted greater antibody responses than offspring of unvaccinated mothers. Similarly, but focussing on shorter time-scales, Grindstaff *et al*. (2006) found that maternal vaccination immediately before egg-laying increased endogenous antibody production but not baseline antibody titres in young pied flycatcher (*Ficedula hypoleuca*) chicks.

Maternal antibody transfer may particularly benefit young offspring whose own immune systems have not yet developed, but has also been suggested to permanently alter offspring immune function (Lemke & Lange 1999; Grindstaff *et al*. 2003). Long-term maternal effects have been demonstrated in laboratory mice (Lemke & Lange 1999), but it is less clear whether substantive maternal effects persist over long time-scales in the wild. Female great tits (*Parus major*) exposed to nest parasites during laying produced offspring that were more likely to recruit locally (Heeb *et al*. 1998). In contrast, effects of maternal *f* on cell-mediated immunity in song sparrow chicks were no longer evident in fledged juveniles (Reid *et al*. 2003) and humoral immunity did not vary with natal nutrition in adult blue tits (*Parus caeruleus*, Råberg *et al*. 2003). Our data suggest that in free-living birds, maternal immune experience can cause substantial long-term variation in immune responsiveness in fully grown offspring, which moreover had hatched seven to nine months after maternal exposure. Since vaccinated parents were no less likely to survive from 2004 to 2005 than unvaccinated parents (*p*>0.4), these patterns are not an artefact of selective vaccination-induced mortality in poor quality parents.

Such long-term consequences of maternal vaccination may represent direct, permanent effects of maternal antibody transfer on offspring immunology (Lemke & Lange 1999; Grindstaff *et al*. 2006). Alternatively, maternal vaccination could influence offspring immunity by altering parental investment in reproduction (and thereby offspring quality, Grindstaff *et al*. 2003). We cannot distinguish these mechanisms definitively. However, vaccinated and unvaccinated song sparrow parents did not differ in reproductive performance in 2005, suggesting that vaccination did not cause major changes in parental investment. Furthermore, in song sparrows, both parents provision chicks (Smith *et al*. 2006). The effect of maternal but not paternal vaccination on offspring immunity may therefore indicate a direct effect of maternal antibody transfer rather than a general consequence of altered parental investment. Finally, we note that since we could not apply a sham injection control given constraints of our wider study, we cannot distinguish whether maternal vaccination caused a general or tetanus-specific increase in offspring immune response. However, given the known specificity of vertebrate humoral immunity (Roitt *et al*. 1998), a tetanus-specific effect seems probable.

Although substantial, the increased tetanus response in offspring of vaccinated mothers was considerably lower than the average secondary antibody response measured in 2005 in seven parents that had also been vaccinated in 2004 (4488±664 units, see also Owen-Ashley *et al*. 2004). Therefore, maternal vaccination did not prompt a full secondary antibody response in offspring (as observed in laboratory mice, Lemke & Lange 1999). However, the fourfold average increase in response observed in offspring of vaccinated females seems likely to be biologically significant (Møller & Saino 2003). Variation in primary humoral immune response therefore substantively reflects long-term inter-generational effects of maternal immune experience in these free-living birds. Such long-term, indirect environmental effects should be incorporated into evolutionary and epidemiological models of host–parasite interactions (Wolf *et al*. 1998; Grindstaff *et al*. 2003).

## Figures and Tables

**Table 1:** Comparison of inbreeding coefficient (*f*) and 2005 age and reproductive performance of song sparrow parents that had and had not been vaccinated in September 2004. (Single vaccinated and unvaccinated mothers were immigrants to Mandarte and of unknown *f*.)

		*n*	*f*	age (years)	lay date (Julian)	total breeding attempts	total eggs	total independent offspring	independent offspring per egg
mothers	unvaccinated	10	0.047±0.01	2.3±0.4	96.7±2.1	2.8±0.2	9.8±0.8	6.4±0.7	0.67±0.1
	vaccinated	11	0.055±0.01	1.6±0.4	96.7±2.8	2.6±0.2	9.1±0.7	5.6±0.8	0.61±0.1
			*t*_17_=0.6	*t*_19_=1.3	*t*_19_=0.1	*t*_19_=0.5	*t*_19_=0.7	*t*_19_=0.8	*t*_19_=0.7
			*p*=0.59	*p*=0.21	*p*=0.91	*p*=0.65	*p*=0.51	*p*=0.42	*p*=0.49
fathers	unvaccinated	13	0.038±0.01	3.3±0.5	96.5±2.5	2.5±0.3	8.2±1.0	4.8±0.7	0.61±0.1
	vaccinated	10	0.047±0.01	2.0±0.4	101.8±4.4	2.3±0.2	8.6±0.7	5.7±0.9	0.65±0.1
			*t*_21_=0.8	*t*_21_=2.0	*t*_21_=1.1	*t*_21_=0.4	*t*_21_=0.4	*t*_21_=0.8	*t*_21_=0.3
			*p*=0.44	*p*=0.06	*p*=0.28	*p*=0.69	*p*=0.73	*p*=0.42	*p*=0.75

**Table 2:** Models relating (*a*) baseline tetanus antibody titres and (*b*) tetanus response in fully-grown song sparrow offspring to parental vaccination. (Terms retained in final models are indicated in bold. ‘Family’ effects were not significant.)

	maternal vaccination	paternal vaccination	inter-sample period	inter-sample period ^2^	offspring *f*	paternal *f*	maternal *f*	offspring sex	final model
baseline	*F*=0.1	*F*<0.1	—	—	*F*=1.1	*F*=0.9	*F*<0.1	*F*<0.1	—
antibody titre	*p*=0.77	*p*=0.89	—	—	*p*=0.29	*p*=0.34	*p*=0.77	*p*=0.80	—
tetanus	***F*=11.8**	*F*=0.6	***F*=10.3**	***F*=9.5**	***F*=5.1**	***F*=3.3**	*F*=0.1	*F*<0.1	***F*_5,45_=6.7**
response	***p*=0.001**	*p*=0.43	***p*=0.003**	***p*=0.004**	***p*=0.030**	***p*=0.078**	*p*=0.75	*p*=0.95	***p*<0.001**
	***B*=−0.6**	*B*=−0.1	***B*=3.5**	***B*=−0.2**	***B*=−4.6**	***B*=5.5**			***R*^2^=0.39**
	***η*^2^=0.23**	*η*^2^=0.02							

## Supplementary Material

Measurement of tetanus antibody titres and tetanus response in song sparrows (*Melospiza melodia*) Here we provide a brief description of the methods used to quantify baseline and post-vaccination tetanus antibody titres in song sparrow offspring
