# Supplementary material for: Long-term maternal effect on offspring immune response in song sparrows Melospiza melodia
Source: Biol Lett. 2006 Sep 26;2(4):573–6. doi: 10.1098/rsbl.2006.0544 (PMC1834015; doi:10.1098/rsbl.2006.0544)
Supplement: Measurement of tetanus antibody titres and tetanus response in song sparrows (Melospiza melodia) — Here we provide a brief description of the methods used to quantify baseline and post-vaccination tetanus antibody titres in song sparrow offspring [file rsbl20060544s21.pdf]

Electronic supplementary material

Measurement of tetanus antibody titres and tetanus response

Song sparrows were mist-netted, biometrics were recorded and ≤100μl blood was collected by brachial venipuncture. Individuals were vaccinated with 70μl human diphtheria-tetanus vaccine (DTV, 2Lf diphtheria toxoid, 5Lf tetanus toxoid adsorbed in aluminium phosphate, Aventis Pasteur) in the pectoral muscle and released. Since primary antibody responses peak 9-15 days after vaccination in song sparrows (Owen-Ashley *et al*. 2004), we attempted to recapture sparrows 10-12 days after vaccination. Recaptured sparrows were blood sampled as before and released. Since we could not control the exact timing of recapture of free-flying individuals, we blood sampled all individuals recaptured 8-14 days after vaccination and subsequently controlled statistically for inter-sample period (as a linear and quadratic covariate, Reid *et al*. 2006). Blood samples were placed immediately on ice and centrifuged for four minutes at 3000rpm within five hours. Plasma was separated off, stored buried in ice and frozen at -20°C within 72 hours. Enzyme-linked immunosorbent assays (ELISAs) were subsequently used to quantify tetanus antibody titres in pre- and post-vaccination plasma samples. We did not analyse diphtheria antibody titre data because the primary antibody response to diphtheria toxoid is low in song sparrows (see Owen-Ashley *et al*. 2004; Reid *et al*. 2006). Protocols followed those previously developed for song sparrows and other passerines (Hasselquist *et al*. 1999, 2001; Owen-Ashley *et al*. 2004). ELISA plates held an individual's pre- and post-vaccination plasma samples in duplicate. Titres were standardised for any among-plate variation in conditions or reagents within each year by reference to serially diluted standard samples that were included on each plate.

Individual sexes were determined by observing breeding behaviour in 2006 and by PCR amplification of sex-linked CHD1 genes. Individual and parental inbreeding coefficients (f) were calculated directly from the substantial pedigree information that exists for the Mandarte song sparrow population (Keller 1998; Reid *et al*. 2006). Unvaccinated parents that were and were not captured during September 2004 did not differ with respect to age, f or reproductive performance in 2005 (all $p > 0.25$). Final models remained qualitatively identical when parent age was included as a covariate.

Hasselquist, D., Marsh, J.A., Sherman, P.W. & Wingfield, J.C. 1999 Is avian humoral immunocompetence suppressed by testosterone? *Behav. Ecol. Sociobiol.* **45**, 167-175.

Hasselquist, D., Wasson, M.F. & Winkler, D.W. 2001 Humoral immunocompetence correlates with date of egg-laying and reflects work load in female tree swallows. *Behav. Ecol.* **12**, 93-97.

Keller, L.F. 1998 Inbreeding and its fitness effects in an insular population of song sparrows (*Melospiza melodia*). *Evolution* **52**, 240-250.

Owen-Ashley, N.T., Hasselquist, D. & Wingfield, J.C. 2004 Androgens and the immunocompetence handicap hypothesis: unravelling direct and indirect pathways of immunosuppression in song sparrows. *Am. Nat*. **164**, 490-505.

Reid, J.M., Arcese, P., Keller, L.F., Elliott, K., Sampson, L., & Hasselquist, D. 2006. Inbreeding effects on immune response in free-living song sparrows (*Melospiza melodia*). *Proc. R. Soc. Lond*. B *in press*.
